# Severe Gastrointestinal Hemorrhage due to Monkeypox Virus-Associated Proctocolitis

**DOI:** 10.1155/2023/9981631

**Published:** 2023-07-17

**Authors:** Sobaan Taj, Chris Austin, Azhar Hussain, Maryam Salma Babar, Harshavardhan Sanekommu, Steven Imburgio, Anmol Johal, Alejandro CruzPonce, Apurva Vedire, Edward Liu

**Affiliations:** ^1^Department of Medicine, Jersey Shore University Medical Center, Neptune City, New Jersey, USA; ^2^Department of Medicine, St. George's University School of Medicine, True Blue, Grenada; ^3^Ameer ud Din Medical College, Lahore, Pakistan; ^4^Cleveland Clinic, Abu Dhabi, UAE

## Abstract

Monkeypox is primarily a painful cutaneous disease with occasional systemic manifestations. Monkeypox is transmitted predominantly through close physical contact and occasionally sexual contact. The first case was reported in the United States on May 17, 2022, in a recent monkeypox worldwide outbreak. We present a case of severe gastrointestinal bleeding as an atypical manifestation of monkeypox infection in a 40-year-old male with HIV. In our case, monkeypox-induced proctocolitis progressed to severe rectal bleeding requiring one unit of packed red blood cells transfusion despite one week of tecovirimat (TPOXX) therapy. So, patients should be educated about the possibility of unusual complications of monkeypox infection, i.e., bleeding in immunocompromised hosts.

## 1. Introduction

Monkeypox virus (MPXV) is a member of the orthopoxvirus family that is endemic to the tropical rainforests of Central and Western Africa. In comparison to smallpox, monkeypox is less contagious and causes milder symptoms. In May 2022, a new outbreak of monkeypox was first reported in Europe and then worldwide, making it a global public health emergency on July 23, 2022 [1]. The United States reported its first case on May 17, 2022 [2]. In this report, we present a case of severe gastrointestinal bleeding as an atypical manifestation of monkeypox infection.

## 2. Case Presentation

**Figure 1:**
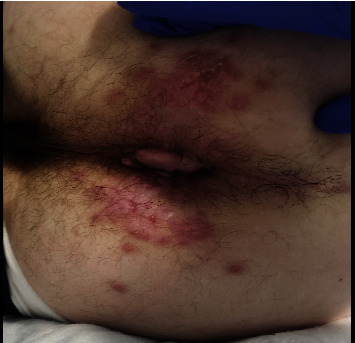
Monkeypox-induced perianal lesions. These painful perianal lesions were the initial manifestation of the monkeypox virus infection.

A 40-year-old male with a past medical history of HIV (CD4 count = 537 cells/mm^**3**^, viral load <20 copies/mL) on antiretroviral therapy (bictegravir-emtricitabine-tenofovir alafenamide), hemorrhoids, and a recently diagnosed monkeypox virus infection, presented to the hospital with hematochezia. The patient has no history of inflammatory bowel disease (IBD), diverticulosis, colonic polyps, or any prior gastrointestinal bleeding. The patient was tested for the monkeypox virus two weeks prior to this admission after developing painful anal lesions (Figure 1). The PCR test of anal and rectal swabs for monkeypox virus (MPXV) was positive, and he was started on oral tecovirimat (TPOXX) 600 mg twice daily for 14 days.

**Figure 2:**
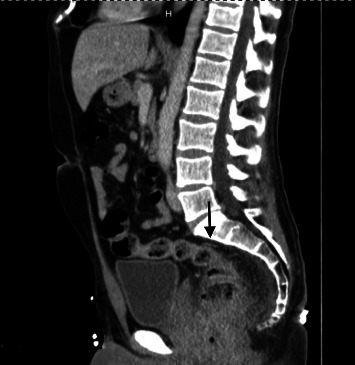
CT angiography (CTA) of the abdomen and pelvis. CT angiography revealed circumferential wall thickening with significant inflammatory changes at the level of the distal rectum and anus, indicating proctocolitis (black arrow). Small rounded hypodensities, adjacent to the distal rectum measuring 11 and 14 mm, were suspicious for rectal abscesses.

Prior to this hospitalization, the patient had recurrent episodes of bloody diarrhea at home for the last two weeks. Despite one-week tecovirimat (TPOXX) therapy, MPXV-induced proctitis progressed to severe rectal bleeding requiring transfusion. He had an episode of syncope and woke up on the bathroom floor covered in diarrhea and blood. Then, he called 911 (Universal Emergency Number) and was brought to the emergency department (ED). In ED, the patient was tachycardic and had a hemoglobin of 9.9 (10^3^ g/*µ*L) which was much lower than his baseline hemoglobin of 15.1 (10^3^ g/*µ*L) recorded two weeks ago. The patient's hemoglobin dropped further to 7.0 (10^3^ g/*µ*L) during this admission. CT angiography (CTA) abdomen and pelvis (Figure 2) revealed circumferential wall thickening with significant inflammatory changes at the level of the distal rectum and anus indicating proctocolitis. Two small, round hypodensities were observed near the distal rectum, with measurements of 11 mm and 14 mm. These findings raised suspicion of rectal abscesses.

Infectious diseases and gastroenterology were consulted and taken on board for further evaluation. Infectious diseases recommended continuing with Tecovirimat (TPOXX) and attributed bloody diarrhea secondary to monkeypox-induced proctitis. He had started on broad-spectrum antibiotics given the possibility of rectal abscesses. His hemoglobin stabilized after 1 unit of packed red blood cells (PRBC), and no further episodes of bleeding occurred, so no endoscopic procedure was performed. The patient was discharged eventually to finish his 14-day course of tecovirimat (TPOXX) and was followed up after two weeks with a resolution of anal lesions and no further episodes of gastrointestinal bleeding with stable and rising hemoglobin levels.

## 3. Discussion

Monkeypox primarily causes painful skin lesions but can also result in systemic symptoms. The disease is transmitted through close physical contact, and MPXV has been detected in semen samples, suggesting a potential for sexual transmission [3]. As a result, oral and rectal lesions are frequently observed in monkeypox cases. While most patients can be treated as outpatients, some may require hospitalization for complications such as severe pain, dehydration, or sepsis.

Severe anorectal pain is a common reason for hospitalization, and proctitis and/or proctalgia can occur in up to 22–37% of cases. Diagnosis can typically be made based on clinical suspicion, with confirmation via PCR of anal or rectal swabs. Imaging is not typically required, but CT scans can be useful for diagnosing monkeypox-induced proctitis without the need for invasive procedures such as colonoscopy or sigmoidoscopy [4]. However, in some cases, severe proctitis with anal and rectal ulcerations has been observed during proctoscopies [5].

The mechanism by which MPXV affects hemostasis remains poorly understood. However, it is postulated to be like Ebola, Dengue, and Hanta virus [6]. Specifically, the infected cells lose their capacity to produce type 1 interferon, leading to lymphocytic death. The poor function of dendritic cells can disrupt the immune system and heighten vascular permeability, which is further exacerbated by cytokine release from infected macrophages. Consequently, the viruses can instigate dysfunction of visceral cells, platelet impairment, and coagulopathy, ultimately culminating in intravascular coagulation and hemorrhage [7, 8].

This case demonstrates the possibility of hemodynamically significant bleeding occurring as a result of monkeypox-induced proctocolitis. The exact cause of the bleeding is unclear, but it could be due to severe proctitis damaging the hemorrhoidal veins and worsening rectal ulcerations. While there are several possible causes of lower gastrointestinal bleeding in immunocompromised patients, including drug induced colitis, CMV colitis, HSV colitis, HIV-associated colitis, and diverticulosis, the patient's young age, well-controlled HIV infection, lack of previous history of GI bleeding, and presence of perineal lesions all suggest that monkeypox-induced proctocolitis was the likely cause.

Drug-induced colitis can be considered high in the differential as it can present with hematochezia [9]. The symptoms initially involve nonbloody diarrhea, which can progress to hematochezia. These symptoms may be caused by inflammation of the colonic wall or pancreatic failure resulting in steatorrhea [9]. Our patient has been experiencing persistent bloody bowel movements for two weeks without any reported episodes of diarrhea. Furthermore, the patient's only new medication prior to the onset of symptoms is tecovirimat. A thorough review of the literature did not reveal any association between this medication and colitis.

Given the risk of potential exposure to healthcare workers, colonoscopy was not performed, but the gastroenterology team was available in case of further bleeding. Although there have been reports of other viral-induced proctitis [10, 11] resulting in severe bleeding, such as CMV-induced proctitis [10], further studies are needed to establish a definitive association between monkeypox infection and proctocolitis-related gastrointestinal hemorrhage.

To conclude, our case report highlights a rare but severe complication of monkeypox infection, where a patient with HIV developed severe rectal bleeding despite being on TPOXX for a week. It is important to educate patients, especially those who are immunocompromised, about the potential for atypical complications of monkeypox infection, including bleeding and sepsis. Further studies are necessary to establish an association between monkeypox infection and proctocolitis-related gastrointestinal hemorrhage.

## Data Availability

The data used to support the findings of this study are available from the corresponding author upon reasonable request.

## References

[1] Vaughan A. M., Cenciarelli O., Colombe S. (2022). A large multi-country outbreak of monkeypox across 41 countries in the WHO European Region, 7 March to 23 August 2022. *Euro Surveillance*.

[2] Basgoz N., Brown C. M., Smole S. C. (2022). Case 24-2022: a 31-year-old man with perianal and penile ulcers, rectal pain, and rash. *New England Journal of Medicine*.

[3] Thornhill J. P., Barkati S., Walmsley S. (2022). Monkeypox virus infection in humans across 16 countries april-june 2022. *New England Journal of Medicine*.

[4] Messina M. D., Wolf E. L., Kanmaniraja D., Alpert P. L., Ricci Z. J. (2022). Imaging features of anorectal proctitis in monkeypox infection. *Clinical Imaging*.

[5] Pfäfflin F., Wendisch D., Scherer R. (2022). Monkeypox in-patients with severe anal pain. *Infection*.

[6] Iba T., Levy J. H., Levi M. (2022). Viral-induced inflammatory coagulation disorders: preparing for another epidemic. *Thrombosis and Haemostasis*.

[7] Basler C. F. (2017). Molecular pathogenesis of viral hemorrhagic fever. *Seminars in Immunopathology*.

[8] Marietta M., Coluccio V., Luppi M. (2022). Monkeypox outbreak: after COVID-19, another challenge for the hemostatic system?. *Internal and emergency medicine*.

[9] Jevtic D., Dumic I., Nordin T. (2021). Less known gastrointestinal manifestations of drug reaction with eosinophilia and systemic symptoms (dress) syndrome: a systematic review of the literature. *Journal of Clinical Medicine*.

[10] Alam I., Shanoon D., Alhamdani A., Boyd A., Griffiths A. P., Baxter J. N. (2007). Severe proctitis, perforation, and fatal rectal bleeding secondary to cytomegalovirus in an immunocompetent patient: report of a case. *Surgery Today*.

[11] Kim R., Sigle G. (2015). Sexually transmitted proctitis. *Clinics in Colon and Rectal Surgery*.

